# Trifluoperazine induces cellular apoptosis by inhibiting autophagy and targeting NUPR1 in multiple myeloma

**DOI:** 10.1002/2211-5463.12960

**Published:** 2020-08-31

**Authors:** Anmao Li, Xuanxin Chen, Zizi Jing, Jianbin Chen

**Affiliations:** ^1^ Department of Hematology the First Affiliated Hospital of Chongqing Medical University Yuzhong China

**Keywords:** apoptosis, autophagy, multiple myeloma, nuclear protein 1, trifluoperazine

## Abstract

Multiple myeloma (MM) is the second most common hematologic malignancy of immunoglobulin‐secreting plasma cells. Recent modern combination therapies have improved survival rates, but many patients develop resistance to novel drugs, leading to relapse. Trifluoperazine (TFP), a typical antipsychotic drug, has been reported to exert antitumor effects by targeting various pathways. Thus far, the role of TFP in MM has not been elucidated. In the current study, we demonstrated that TFP inhibited cell growth and autophagy activity but induced apoptosis of U266 and RPMI 8226 MM cells. Furthermore, cotreatment of these cell lines with TFP and rapamycin, a potent autophagy inducer, reduced cell apoptosis compared with TFP treatment alone. We also found that TFP inhibited nuclear protein 1 (NUPR1) expression. In the presence of TFP, cells stably overexpressing NUPR1 showed a higher viability than cells treated with the nonspecific control. Autophagy suppression and apoptosis induction caused by TFP were also reversed in MM cells upon NUPR1 overexpression. Overall, our results indicate that in the context of MM, TFP targets NUPR1, inhibiting cell growth and inducing apoptosis by autophagy inhibition. Our results could contribute toward efforts for the development of more effective therapies for MM to be tested in future clinical trials.

## Graphical abstract

Multiple myeloma is the second most common hematological malignancy. Trifluoperazine (TFP), an antipsychotic drug, has been reported to exert antitumor effects. Here, our results indicate that TFP targets NUPR1, inhibiting cell growth and inducing apoptosis by autophagy inhibition in multiple myeloma cells. This study provides more directions for the treatment for multiple myeloma.

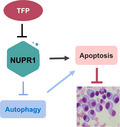

**Abbreviations**
c‐caspase 3: cleaved caspase 3
LV: lentiviral vectors
MM: multiple myeloma
NC: nonspecific control
NUPR1: nuclear protein
RAPA: rapamycin
SD: standard deviation
TFP: trifluoperazine

Multiple myeloma (MM), accounting for more than 17% of all hematologic malignancies, is a destructive and fatal cancer characterized by the neoplastic proliferation of plasma cell clones [1]. Although modern therapies for MM include autologous stem cell transplantation and new chemotherapeutic agents such as bortezomib and lenalidomide, MM is still incurable [2]. It often becomes a recurrent or refractory disease, and drug resistance hinders the management of MM. The repurposing of off‐patent drugs to treat cancer, alone or in combination with other approved drugs, has become an attractive therapeutic strategy for MM.

Trifluoperazine (TFP) is a dopamine D2 receptor inhibitor and a phenothiazine derivative that has been used for many years as an antipsychotic drug [3]. TFP treatment impairs cell viability and induces mitochondria‐mediated intrinsic apoptosis and autophagy [4, 5, 6]. It has thus been employed as an alternative therapeutic strategy for colorectal cancer, glioblastoma, and urothelial carcinoma. Various TFP derivatives, such as ZZW‐115, have been developed that exhibited improved antitumor efficacy and reduced toxicity [7, 8]. Although TFP was identified by bioinformatics analysis as one of the most promising agents for MM treatment [9], its role in MM has not yet been reported.

Nuclear protein 1 (NUPR1) is a recognized transcriptional regulator that controls the stress response, autophagy, and apoptosis in multiple malignancies [7, 10, 11]. We previously discovered that NUPR1 was highly expressed in the bone marrow of MM patients. Silencing it induced autophagy‐mediated apoptosis of MM cells through the PI3K/AKT/mTOR signaling pathways [12]. Santofimia‐Castano reported that TFP, as well as ZZW‐115, bound to NUPR1 and inhibited its translocation from the cytoplasm to the nucleus [13]. It subsequently induced programmed cell death in pancreatic cancer [7]. However, the interaction between TFP and NUPR1 in MM remains undetermined.

In the current study, we investigated the role of TFP and its association with NUPR1, with the focus on cell death. We show that TFP inhibits growth and induces apoptosis by inhibiting autophagy and targeting NUPR1 in MM cells. An understanding of the mechanism by which TFP modulates autophagy and apoptosis is significant for its further development as a clinically useful therapeutic agent for MM.

## Materials and methods

### Cell culture

The MM cell lines (U266 and RPMI 8226) were donated by J. Hou of the Affiliated Renji Hospital of Shanghai Jiaotong University (Shanghai, China). Cells were maintained in RPMI‐1640 medium (Gibco, New York, NY, USA), supplemented with 10% fetal bovine serum (PAN, Germany), and were cultured at 37 °C in an atmosphere of 5% CO_2_ in air.

### Cell transfection and agents

NUPR1 lentiviral vectors (LVs) and nonspecific control (NC) LVs were synthesized and produced as previously described by Shanghai GeneChem Co., Ltd. (Shanghai, China) [14]. We seeded approximately 5 × 10^4^ cells per well in a 24‐well plate, and then added the corresponding LVs for a multiplicity of infection of 100. After 12 h, we removed the LVs. The transfected cells were selected by Solarbio (Beijing, China). Transfection efficiency was assessed by detecting green fluorescent protein using fluorescence microscopy and flow cytometry.

Trifluoperazine (purity > 99.0%) and rapamycin were purchased from MedChemExpress (Monmouth Junction, NJ, USA).

### Cell counting Kit‐8 assay

Cultured MM cells were seeded in 96‐well plates (5 × 10^3^ cells per well), subjected to TFP treatment at different concentrations, and incubated at 37 °C for 24 h. To each well, 10 μL of Cell Counting Kit‐8 reagent (CCK‐8; Bimake, Houston, TX, USA) was added. After incubation at 37 °C for 2 h, the wells were assessed with a microplate reader (Thermo Fisher Scientific, Waltham, MA, USA) at an absorbance of 450 nm.

### Lactate dehydrogenase assay

We used a lactate dehydrogenase (LDH) cytotoxicity test kit (Dojindo, Tokyo, Japan) to detect released LDH from the cytosol. After treatment with 30 μm TFP for 24 h, approximately 5 × 10^3^ MM cells of each group were inoculated per well of a 96‐well plate. We added 50 μL of medium containing the test substance that was adjusted to the desired concentration and incubated the plate at 37 °C for 1 h in a CO_2_ incubator. Then, we added 10 μL of lysis buffer to each well of the high control, and the plate was incubated once more at 37 °C for 30 min. After adding 100 μL of working solution to each well, we incubated the plate at room temperature for 30 min and then added 50 μL of stop solution. The absorbance at 490 nm was measured, and the relative cytotoxicity was calculated from the amount of released LDH divided by the amount of total LDH.

### Reverse transcription–quantitative polymerase chain reaction

After treatment with TFP for 24 h, U266 and RPMI 8226 cells were gently centrifuged and collected. RNAiso Plus was used to extract the total RNA according to the manufacturer’s instructions. The cDNA was then synthesized using the Prime Script™ RT reagent kit. Reverse transcription–quantitative real‐time PCR (RT‐qPCR) analysis was performed using a CFX96 Real‐Time PCR System with a SYBR Premix Ex Taq™ II PCR Kit. The primers for *NUPR1* were as follows: forward, 5'‐AGGACTTATTCCCGCTGACTGA‐3' and reverse, 5'‐TGCCGTGCGTGTCTATTTATTG‐3'; *β‐actin*: forward, 5'‐CCACGAAACTACCTTCAACTCC‐3' and reverse, 5'‐GTGATCTCCTTCTGCATCCTGT‐3'. The relative gene expression was analyzed by the 2^−ΔΔCq^ method. All reagents used for RT‐qPCR were purchased from TaKaRa (Dalian, China).

### Western blot analysis

U266 and RPMI 8226 cells were harvested and lysed with cell lysis buffer (poly(N‐isopropylacrylamide) (PIPA) mixed with phenylmethylsulfonyl fluoride (PMSF) at a ratio of 100: 1, Beyotime, Shanghai, China. We then measured the concentration of the extracted protein using a bicinchoninic acid (BCA) assay kit (Beyotime) and balanced the concentration. Proteins in equal amounts (20–30 μg) were added to different wells of pre‐prepared gels, separated by sodium dodecyl sulfate–polyacrylamide gel electrophoresis (SDS/PAGE), and subsequently transferred to polyvinylidene fluoride (PVDF) membranes. After blocking with 5% nonfat milk for 2 h, the PVDF membranes were subject to incubation with the primary antibodies at 4°C overnight. The following primary antibodies were used: rabbit anti‐human NUPR1 antibody (1: 1000, Novus, St Charles, MO, USA); LC3, P62, and cleaved caspase 3 (1 : 1000, Cell Signaling Technology, Boston, MA, USA); and BCL2, BAX, and GAPDH (1 : 1500, Affinity, Milwaukee, WI, USA). The membranes were washed three times and incubated with horseradish peroxidase‐conjugated secondary antibody (1 : 3000, CST) for 1 h. We then washed the membranes again three times. Protein bands were detected by using an enhanced chemiluminescence (ECL) kit (Beyotime) with the FUSION FX imaging analysis system (Vilber Lourmat, France). The relative expression was obtained by dividing the band density of the target protein by the density of glyceraldehyde‐3‐phosphate dehydrogenase (GAPDH) obtained using Fusion software. LC3 analysis was evaluated by the ratio of the band density of LC3 II and LC3 I.

### Flow cytometry

After TFP treatment for 24 h, the seeded approximately 10^6^ MM cells were washed and resuspended in phosphate‐buffered saline (PBS). Cells at different stages of apoptosis were distinguished with Annexin V‐APC/propidium iodide (PI) staining (Syngene, China) following the manufacturer’s instructions. Apoptosis analysis was performed on a FACS flow cytometer with cellquest software (Becton Dickinson, Franklin Lakes, NJ, USA).

### Transmission electron microscopy

Transmission electron microscopy (TEM) was applied to evaluate cell autophagy. Cells treated with 30 μm TFP for 24 h and untreated control cells were fixed in 3% glutaraldehyde in PBS. Fixation in OsO4 was then performed, and the cells were encapsulated in agarose. Collected cells were then dehydrated, embedded, and polymerized in Spurr resin. Ultrathin sections were obtained and double‐stained with uranyl acetate and lead citrate. Grids were observed on a transmission electron microscope (Hitachi, Japan). At least 200 cells from 4 grids were analyzed for each set.

### Statistical analysis

All analyses were performed with GraphPad Prism 7 (San Diego, CA, USA) and SPSS (Chicago, IL, USA) software, with at least three independent experiments that are presented as the mean ± SD. Statistically significant differences between two groups were assessed using an unpaired Student’s *t*‐test. The comparison of three or four groups was performed by analysis of variance (ANOVA). Differences were considered statistically significant when */#*P* < 0.05, **/##*P* < 0.01, and ***P < 0.001.

## Results

### TFP inhibits growth and autophagy but induces apoptosis of MM cells

**Fig. 1:**
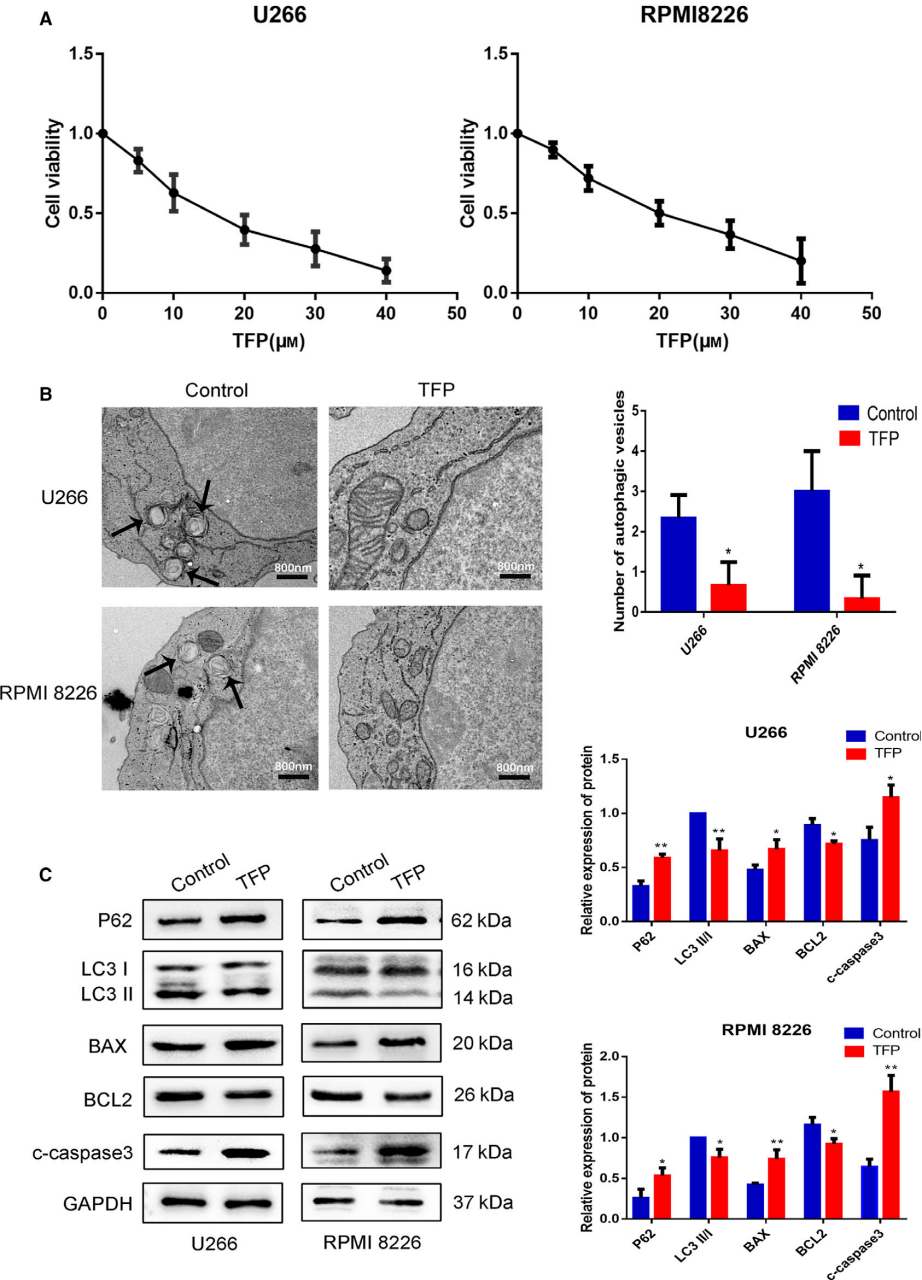
Trifluoperazine inhibited MM cell growth and induced apoptosis *in vitro*. (A) CCK‐8 assay was used to analyze the viability of U266 and RPMI 8226 cells following the treatment of TFP at concentrations of 0, 5, 10, 20, 30, and 40 μm for 24 h (*n* = 3). (B) Representative transmission electron micrographs of the control and TFP‐treated (30 μm for 24 h) groups of MM cell lines (*n* = 3, Student’s *t*‐test). Black arrows point to the autophagosomes. Scale bars: 800 nm. (C) Western blot showed the protein expression of P62, LC3, BAX, BCL2, and c‐caspase 3 in the control and TFP‐treated (30 μm for 24 h) groups (*n* = 3, Student’s *t*‐test). */#P < 0.05; **/##*P* < 0.01. Error bars represent SD.

Considering that TFP plays an inhibitory role in various tumors [5, 15], we treated MM cells with different concentrations of TFP (0, 5, 10, 20, 30, 40 μm) to investigate the role of TFP in MM. After treatment for 24 h, we performed the CCK‐8 assay to evaluate the cell viability. As shown in Fig. 1A, cell viability exhibited a downward trend as TFP concentration increased. The IC_50_ values of TFP were 20 μm for U266 and 25 μm for RPMI 8226 cells. After obtaining this information, we proceeded to determine the effects of TFP on MM cell death.

**Fig. 2:**
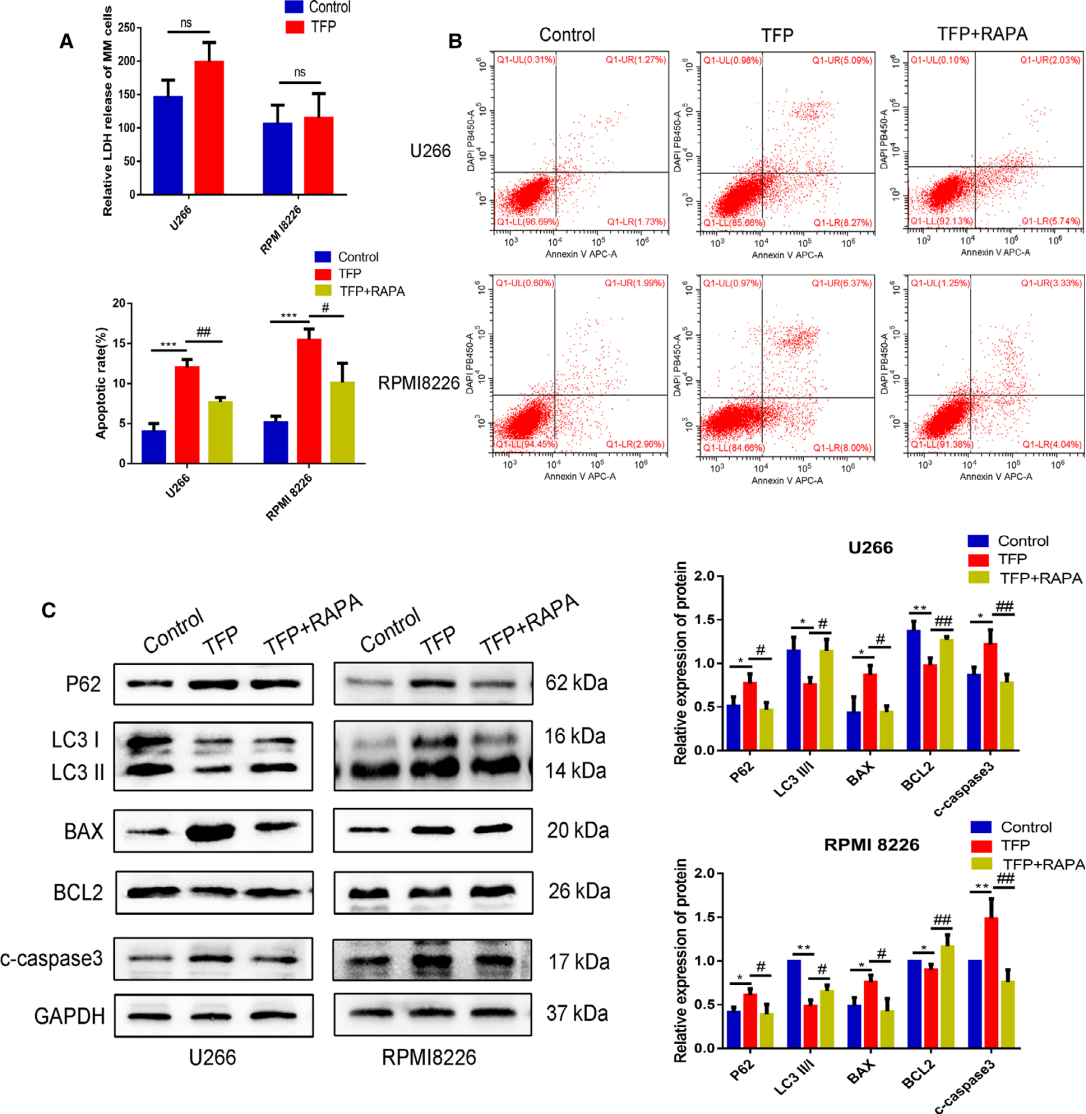
Trifluoperazine induced cellular apoptosis by inhibiting autophagy in U266 and RPMI 8226 cells. (A) LDH release of MM cells treated with TFP (30 μm) for 24 h (*n* = 3, Student’s *t*‐test). (B) Apoptotic rates for each group were evaluated by flow cytometry (*n* = 3, one‐way ANOVA). (C) Western blot was performed to detect the expression of autophagy‐related and apoptosis‐related markers in MM cells following the treatment with TFP (30 μm) alone or cotreatment with TFP (30 μm) and RAPA (100 nm) for 24 h (*n* = 3, one‐way ANOVA). */#*P* < 0.05; **/##*P* < 0.01; ***P < 0.001; ns no significant. Error bars represent SD.

To further study the mechanism of TFP affecting MM cell death, we firstly examined U266 and RPMI 8226 cells as they underwent autophagy after TFP treatment. TEM analysis was performed to assess the autophagy activity by monitoring autophagosomes. We found that the autophagy level was decreased in the TFP‐treated group compared with the control (Fig. 1B). Western blot further confirmed this observation, showing that the ratio of LC3 II/LC3 I was reduced and P62 expression was upregulated (Fig. 1C). The results indicated that the autophagic activity was inhibited in MM cells following treatment with TFP. Secondly, we measured LDH release to evaluate cell death by necrosis in the presence of TFP. There was no significant difference in LDH release (Fig. 2A), suggesting that TFP did not exert pronecrotic effects on U266 or RPMI 8226 cells. Additionally, we investigated the effect of TFP on cell apoptosis. As depicted in Fig. 2B, flow cytometry revealed that the apoptosis rate was higher in the TFP‐treated group than that in the control. Western blot also showed that the expression of antiapoptotic BCL2 was downregulated, and the expression of cleaved caspase 3 and proapoptotic factor BAX was upregulated in the TFP‐treated group (Fig. 1C). Together, these findings suggested that TFP played an integral role in inhibiting growth and autophagy, but induced apoptosis rather than necrosis in MM cells.

### TFP induces cell apoptosis by inhibiting autophagy in MM

Autophagy and apoptosis often affect each other in MM cells. Observing apoptotic changes after autophagy intervention can help distinguish whether autophagy‐mediated apoptosis or autophagic death occurred. To test whether autophagy inhibition caused by TFP was related to cytotoxicity, we cotreated MM cells with TFP and rapamycin, a common autophagy inducer, and detected cell death. Western blot showed that increased expression of autophagy‐related proteins and decreased levels of apoptosis‐related proteins were observed in the cotreatment as compared to the TFP group (Fig. 2C). We also used flow cytometry to observe that cotreatment with TFP and rapamycin downregulated the apoptosis rate compared with TFP treatment alone (Fig. 2B). TFP‐induced apoptosis was significantly reversed by autophagy activation caused by rapamycin. Collectively, we demonstrated that TFP induced apoptosis by inhibiting autophagy in MM cells.

### TFP targets NUPR1 and induces apoptosis by inhibiting autophagy

**Fig. 3:**
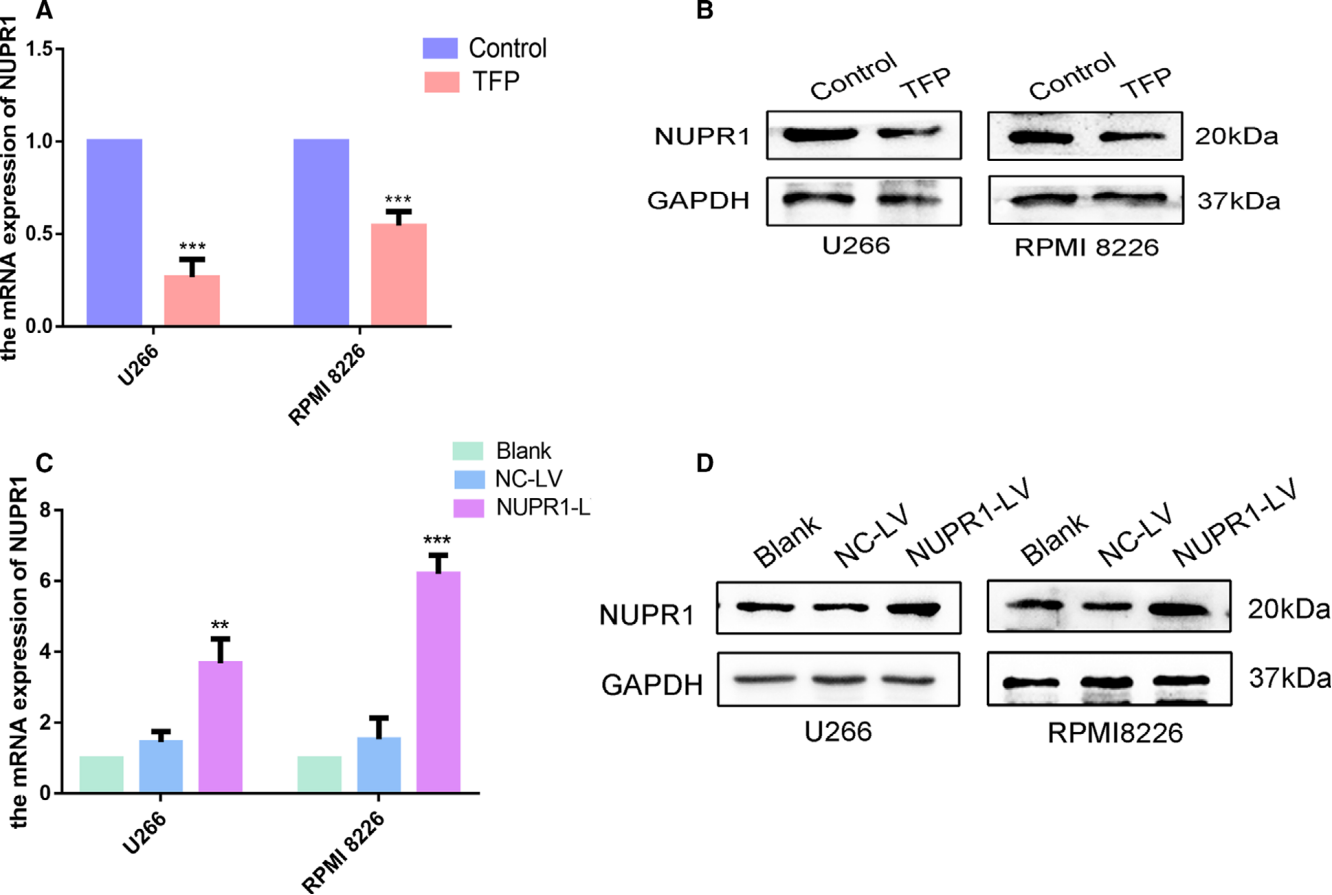
Trifluoperazine suppressed NUPR1 expression in MM cells and stable MM cells overexpressing NUPR1. (A,B) NUPR1 expression at the mRNA and protein levels in MM cells after TFP treatment (30 μm for 24 h) (*n* = 3, Student’s *t*‐test). (C,D) RT‐qPCR and western blot verified the effect of NUPR1 overexpression in the blank, NC‐LV, and NUPR1‐LV groups (*n* = 3, one‐way ANOVA). **P < 0.01; ***P < 0.001. Error bars represent SD.

A study in pancreatic adenocarcinoma has suggested that TFP binds to NUPR1 through the region around amino acid Thr68 [7], indicating that TFP could act as an NUPR1 inhibitor [13]. It was unclear whether NUPR1 was involved in the effects exerted by TFP on MM. We therefore evaluated the expression of NUPR1 in MM cells after TFP treatment. TFP exhibited strong inhibition of NUPR1 expression at both the mRNA and protein levels (Fig. 3A,B), which indicated that NUPR1 might play a role in the effects of TFP on MM cells.

**Fig. 4:**
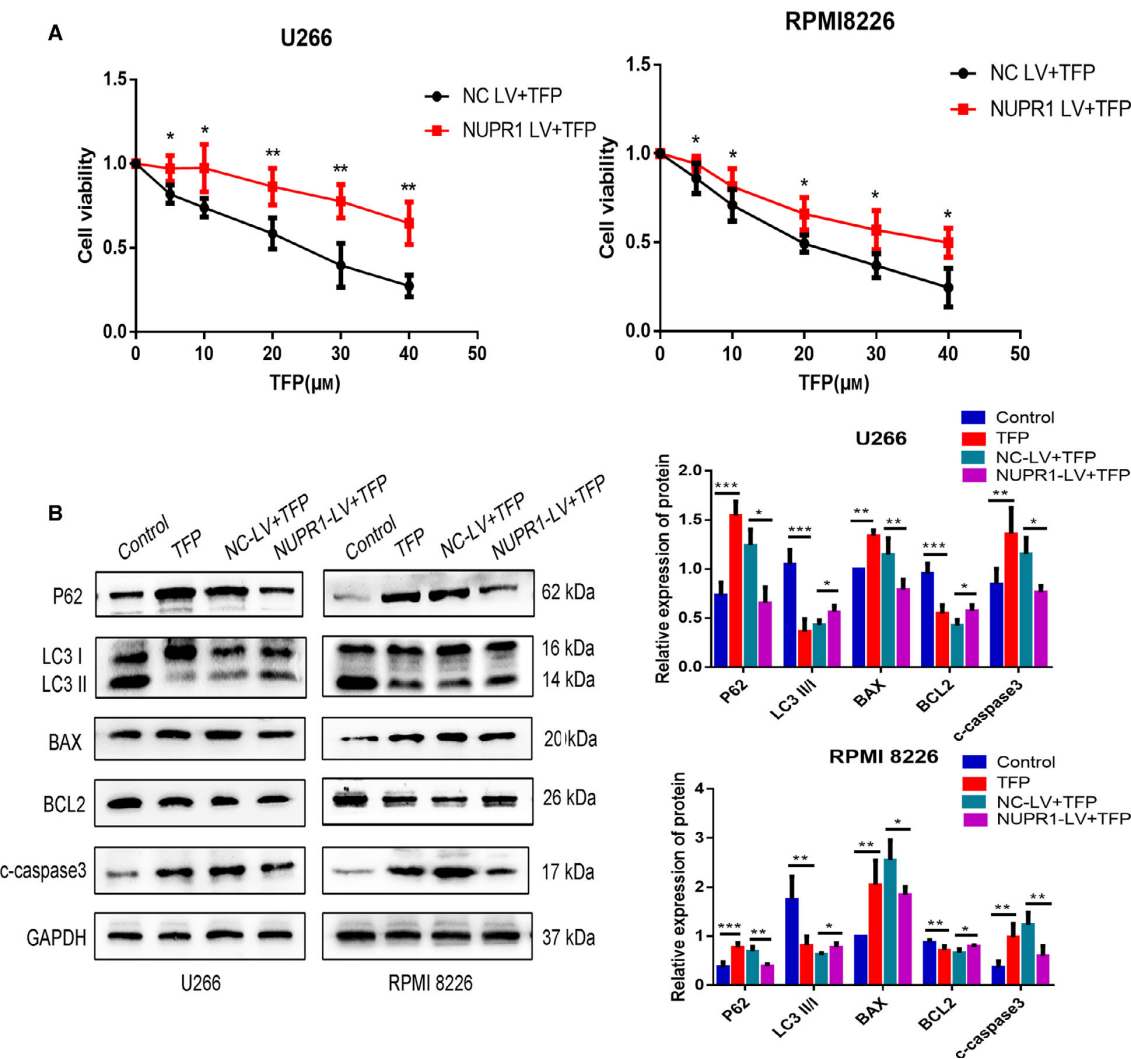
Trifluoperazine induced apoptosis in MM cells by targeting NUPR1 and subsequently inhibiting autophagy. (A) The cell viability of NC‐LV and NUPR1‐LV cells following TFP treatment (30 μm for 24 h) by CCK‐8 assay (*n* = 3, Student’s *t*‐test). (B) The expression of P62, LC3, BAX, BCL2, and c‐caspase 3 of each group was detected by western blot (*n* = 3, one‐way ANOVA). *P < 0.05; **P < 0.01; ***P < 0.001. Error bars represent SD.

To understand whether TFP affected autophagy or apoptosis through regulation of NUPR1, we transfected NC‐LV and NUPR1‐LV into MM cells and constructed stable NUPR1‐overexpressing U266 and RPMI 8226 cells. The overexpressing effects were verified by RT‐qPCR and western blot (Fig. 3C,D). We then treated the NC‐LV and NUPR1‐LV groups with TFP and examined the cell viability by CCK‐8. The results showed that cell viability was restrained after TFP treatment, but the viability of NUPR1‐overexpressing cells was higher than that of the NC‐LV group (Fig. 4A). We also detected the expression of autophagic and apoptotic markers by western blot. The results showed that there was no statistical difference in the expression of the aforementioned proteins between the TFP‐treated group and TFP‐treated NC‐LV group, but the autophagy level was upregulated and apoptosis was downregulated in the TFP‐treated NUPR1‐LV group compared with the TFP‐treated NC‐LV group (Fig. 4B). NUPR1 overexpression reversed the autophagic suppression and cellular apoptosis induction caused by TFP in U266 and RPMI 8226 cells. Thus, we concluded that TFP targeted NUPR1 in MM cells and subsequently induced apoptosis by inhibiting autophagy.

## Discussion

Despite initial clinical success in treating MM with modern combination therapies, many patients eventually relapse. Re‐evaluating old drugs in context with their potential helpful or harmful effects on cancer might identify new alternative compounds for future MM treatment. Here, we focused on TFP, an approved antipsychotic drug. We studied its roles in treating MM *in vitro* for the first time. We found that TFP targeted NUPR1 in U266 and RPMI 8226 cells and then suppressed cell viability and induced apoptosis by inhibiting autophagy.

Studies have shown that TFP plays an anticancer role in several cancer types. It was suggested as a potential agent for tumor growth inhibition in colorectal cancer [5]. This effect was accompanied by induction of apoptosis and autophagy. TFP was also shown to increase radio sensitivity in glioblastoma by impairing homologous recombination [6]. Furthermore, it has been reported that TFP induces downregulation of the P‐gp protein in adriamycin‐resistant L1210 leukemia cells and restores sensitivity to doxorubicin [16]. TFP was also identified as one of the most significant potential agents for MM treatment [10], showing great value in drug development.

In the current study, we focused on the role of TFP in cell death, which consists of necrosis and programmed cell death such as apoptosis, autophagy, and some new types. We found that TFP impaired cell viability and exerted considerable proapoptotic effects on U266 and RPMI 8226 cells. These findings are in contrast with previously reported results, showing that TFP promoted proliferation and reduced calcium‐dependent apoptosis in glioma cells [17]. Additionally, it was reported that TFP promoted cell death by apoptosis and necroptosis in pancreatic cancer [13]. However, our results showed that TFP had no effect on the necrosis of MM cells. These differences could be explained by the supposition that different cells respond differently to the same drug due to their different biological properties.

As the other type of programmed cell death, autophagy is usually a survival mechanism in which cellular homeostasis is maintained through lysosomal recycling of unnecessary and damaged cell components. Plasma cells and MM cells present high autophagy activity, promoting cell survival and leading to drug resistance [18, 19]. During high levels of damage, however, the normal autophagy balance might be disrupted [20]. Autophagy largely depends on stimuli or the cellular environment, and apoptosis may return later, or it may be antagonized or delayed. The two processes could act in a mutually exclusive manner or serve as backup mechanisms for each other, causing cell death. Considering that TFP has been reported to regulate autophagy and autophagic cell death in multiple cancers [5, 6, 8], we investigated the autophagic levels of MM cells following TFP treatment. The observation of the formation of autophagosomes by TEM proved the idea that TFP inhibited the autophagic activity of MM cells. Furthermore, the decreased conversion of LC3 I to LC3 II and accumulation of P62 following TFP treatment indicated the potential disruption of autophagosome formation during autophagy. Moreover, induction of autophagy by rapamycin antagonized the process of cell apoptosis caused by TFP. In MM cells, TFP did not induce autophagy, but rather, it was inhibited. When the process was activated by rapamycin, TFP opposed the mechanism of death induced by the compound. We thus came to the conclusion that TFP caused cellular apoptosis by inhibiting autophagy rather than autophagic death.

NUPR1 is a widely studied molecule, fulfilling different roles in many diseases [7, 21, 22]. It is an intrinsic disorder protein induced by cellular stress and can directly regulate transcription by combining PDGFA, SNAP25, and other gene promoters [21, 23, 24]. NUPR1 thus has a wide range of regulatory effects on cell growth, death, and DNA damage response. We have previously reported that *NUPR1* is overexpressed in MM cells, and its silencing induces autophagy‐mediated apoptosis [25, 26]. Some NUPR1‐related drugs, including TFP and luminespib, have already been shown to have antitumor effects [13, 27]. By binding to NUPR1, TFP inhibits its translocation from the cytoplasm to the nucleus [7]. Through this inhibition, TFP was reported to induce programmed cell death in pancreatic cancer [13]. Moreover, the combination of adeno‐associated virus‐mediated NUPR1 knockdown and TFP induced premature senescence in lung cancer cells [28]. In the current study, we found that TFP attenuated the expression of NUPR1 at both protein and mRNA levels in U266 and RPMI 8226 cells. In the presence of TFP, cells overexpressing NUPR1 exhibited higher cell viability than the nonspecific control. We also showed that NUPR1 overexpression counteracted TFP‐caused autophagy suppression and apoptosis induction. We therefore concluded that TFP regulated cellular autophagy and apoptosis in MM by targeting NUPR1.

In addition, proteasome inhibition by bortezomib, an approved first‐line drug for MM, was shown to increase pancreatic cancer cell sensitivity to TFP [15]. In subsequent experiments, further study should be conducted to determine the association between TFP and bortezomib in MM.

## Conclusion

In summary, our findings show that TFP exhibits significant antitumor effects on MM cells treated *in vitro*. Our study elucidates the behavior of TFP, which induces cellular apoptosis by inhibiting autophagy in MM, at least in part by targeting NUPR1. We provide a proof‐of‐concept study for the repurposing of TFP as a novel therapeutic agent for MM.

## Author contributions

Anmao Li and Xuanxin Chen contributed equally to this work and should be considered cofirst authors. Anmao Li designed the study, performed the present work, and wrote the paper. Xuanxin Chen designed the research, analyzed the results, and revised the paper. Zizi Jing participated in conducting the experiments. Jianbin Chen provided assistance for the coordination of the study, reviewed the manuscript, and provided financial support. All the authors approved the final version of the paper.

## Conflict of interest

The authors declare no conflict of interest.

## Data Availability

All data generated or analyzed during this study are available from the corresponding author upon reasonable request.

## References

[1] Kumar SK , Callander NS , Alsina M , Atanackovic D , Biermann JS , Castillo J , Chandler JC , Costello C , Faiman M , Fung HC *et al* (2018) NCCN guidelines insights: multiple myeloma, version 3.2018. J Natl Compr Canc Netw 16, 11–20. 29295877 10.6004/jnccn.2018.0002

[2] Gay F , Engelhardt M , Terpos E , Wasch R , Giaccone L , Auner HW , Caers J , Gramatzki M , van de Donk N , Oliva S *et al* (2018) From transplant to novel cellular therapies in multiple myeloma: European Myeloma Network guidelines and future perspectives. Haematologica 103, 197–211. 29217780 10.3324/haematol.2017.174573 PMC5792264

[3] Jaszczyszyn A , Gasiorowski K , Swiatek P , Malinka W , Cieslik‐Boczula K , Petrus J and Czarnik‐Matusewicz B (2012) Chemical structure of phenothiazines and their biological activity. Pharmacol Rep 64, 16–23. 22580516 10.1016/s1734-1140(12)70726-0

[4] Kuo KL , Liu SH , Lin WC , Hsu FS , Chow PM , Chang YW , Yang SP , Shi CS , Hsu CH , Liao SM *et al* (2019) Trifluoperazine, an antipsychotic drug, effectively reduces drug resistance in Cisplatin‐resistant urothelial carcinoma cells via suppressing Bcl‐xL: an in vitro and in vivo study. Int J Mol Sci 20, 3218. 10.3390/ijms20133218 PMC6651283 31262032

[5] Qian K , Sun L , Zhou G , Ge H , Meng Y , Li J , Li X and Fang X (2019) Trifluoperazine as an alternative strategy for the inhibition of tumor growth of colorectal cancer. J Cell Biochem 120, 15756–15765. 31081173 10.1002/jcb.28845

[6] Zhang X , Xu R , Zhang C , Xu Y , Han M , Huang B , Chen A , Qiu C , Thorsen F , Prestegarden L *et al* (2017) Trifluoperazine, a novel autophagy inhibitor, increases radiosensitivity in glioblastoma by impairing homologous recombination. J Exp Clin Cancer Res 36, 118. 28870216 10.1186/s13046-017-0588-z PMC5584019

[7] Santofimia‐Castano P , Xia Y , Peng L , Velazquez‐Campoy A , Abian O , Lan W , Lomberk G , Urrutia R , Rizzuti B , Soubeyran P *et al* (2019) Targeting the stress‐induced protein NUPR1 to treat pancreatic adenocarcinoma. Cells 8, 1453. 10.3390/cells8111453 PMC6912534 31744261

[8] Wu CH , Bai LY , Tsai MH , Chu PC , Chiu CF , Chen MY , Chiu SJ , Chiang JH and Weng JR (2016) Pharmacological exploitation of the phenothiazine antipsychotics to develop novel antitumor agents‐A drug repurposing strategy. Sci Rep 6, 27540. 27277973 10.1038/srep27540 PMC4899727

[9] Zhu FX , He YC , Zhang JY , Wang HF , Zhong C and Wang XT (2019) Using Prognosis‐related gene expression signature and connectivity map for personalized drug repositioning in multiple myeloma. Med Sci Monitor 25, 3247–3255. 10.12659/MSM.913970 PMC6510057 31048671

[10] Neira JL , Correa J , Rizzuti B , Santofimia‐Castano P , Abian O , Velazquez‐Campoy A , Fernandez‐Megia E and Iovanna JL (2019) Dendrimers as competitors of protein‐protein interactions of the intrinsically disordered nuclear chromatin protein NUPR1. Biomacromol 20, 2567–2576. 10.1021/acs.biomac.9b00378 31181156

[11] Shiraki M , Xu X , Iovanna JL , Kukita T , Hirata H , Kamohara A , Kubota Y , Miyamoto H , Mawatari M and Kukita A (2019) Deficiency of stress‐associated gene Nupr1 increases bone volume by attenuating differentiation of osteoclasts and enhancing differentiation of osteoblasts. FASEB J 33, 8836–8852. 31067083 10.1096/fj.201802322RR

[12] Li A , Li X , Chen X , Zeng C , Wang Z , Li Z and Chen J (2020) NUPR1 silencing induces autophagy‐mediated apoptosis in multiple myeloma cells through the PI3K/AKT/mTOR pathway. DNA Cell Biol 39, 368–378. 31971825 10.1089/dna.2019.5196

[13] Santofimia‐Castano P , Xia Y , Lan W , Zhou Z , Huang C , Peng L , Soubeyran P , Velazquez‐Campoy A , Abian O , Rizzuti B *et al* (2019) Ligand‐based design identifies a potent NUPR1 inhibitor exerting anticancer activity via necroptosis. J Clin Invest. 129, 2500–2513. 30920390 10.1172/JCI127223 PMC6546470

[14] Scherr M and Eder M (2002) Gene transfer into hematopoietic stem cells using lentiviral vectors. Curr Gene Ther 2, 45–55. 12108973 10.2174/1566523023348237

[15] Huang C , Lan W , Fraunhoffer N , Meilerman A , Iovanna J and Santofimia‐Castano P (2019) Dissecting the anticancer mechanism of trifluoperazine on pancreatic ductal adenocarcinoma. Cancers (Basel) 11, 1869. 10.3390/cancers11121869 PMC6966621 31769431

[16] Shin SY , Choi BH , Kim JR , Kim JH and Lee YH (2006) Suppression of P‐glycoprotein expression by antipsychotics trifluoperazine in adriamycin‐resistant L1210 mouse leukemia cells. Europ J Pharm Sci 28, 300–306. 10.1016/j.ejps.2006.03.002 16707254

[17] Wen Y , Zhang Y , Li J , Luo F , Huang Z and Liu K (2018) Low concentration trifluoperazine promotes proliferation and reduces calcium‐dependent apoptosis in glioma cells. Sci Rep 8, 1147. 29348654 10.1038/s41598-018-19413-y PMC5773581

[18] Yun Z , Zhichao J , Hao Y , Ou J , Ran Y , Wen D and Qun S (2017) Targeting autophagy in multiple myeloma. Leuk Res 59, 97–104. 28599191 10.1016/j.leukres.2017.06.002

[19] Milan E , Fabbri M and Cenci S (2016) Autophagy in plasma cell ontogeny and malignancy. J Clin Immunol 36 (Suppl 1), 18–24. 26984755 10.1007/s10875-016-0254-9 PMC4891390

[20] Roos WP , Thomas AD and Kaina B (2016) DNA damage and the balance between survival and death in cancer biology. Nat Rev Cancer 16, 20–33. 26678314 10.1038/nrc.2015.2

[21] Mu Y , Yan X , Li D , Zhao D , Wang L , Wang X , Gao D , Yang J , Zhang H , Li Y *et al* (2018) NUPR1 maintains autolysosomal efflux by activating SNAP25 transcription in cancer cells. Autophagy 14, 654–670. 29130426 10.1080/15548627.2017.1338556 PMC5959327

[22] Narzt MS , Nagelreiter IM , Oskolkova O , Bochkov VN , Latreille J , Fedorova M , Ni Z , Sialana FJ , Lubec G , Filzwieser M *et al* (2019) A novel role for NUPR1 in the keratinocyte stress response to UV oxidized phospholipids. Redox Biol 20, 467–482. 30466060 10.1016/j.redox.2018.11.006 PMC6243031

[23] Urrutia R , Velez G , Lin M , Lomberk G , Neira JL and Iovanna J (2014) Evidence supporting the existence of a NUPR1‐like family of helix‐loop‐helix chromatin proteins related to, yet distinct from, AT hook‐containing HMG proteins. J Mol Model 20, 2357. 25056123 10.1007/s00894-014-2357-7 PMC4139591

[24] Chen CY , Wu SM , Lin YH , Chi HC , Lin SL , Yeh CT , Chuang WY and Lin KH (2019) Induction of nuclear protein‐1 by thyroid hormone enhances platelet‐derived growth factor A mediated angiogenesis in liver cancer. Theranostics 9, 2361–2379. 31149049 10.7150/thno.29628 PMC6531305

[25] Li A , Li X , Chen X , Zeng C , Wang Z , Li Z and Chen J . (2020) NUPR1 Silencing Induces Autophagy‐Mediated Apoptosis in Multiple Myeloma Cells Through the PI3K/AKT/mTOR Pathway. DNA Cell Biol 39, 368–378. 31971825 10.1089/dna.2019.5196

[26] Zeng C , Li X , Li A , Yi B , Peng X , Huang X and Chen J (2018) Knockdown of NUPR1 inhibits the growth of U266 and RPMI8226 multiple myeloma cell lines via activating PTEN and caspase activation‐dependent apoptosis. Oncol Rep 40, 1487–1494. 30015974 10.3892/or.2018.6544

[27] Augello G , Emma MR , Cusimano A , Azzolina A , Mongiovi S , Puleio R , Cassata G , Gulino A , Belmonte B , Gramignoli R *et al* (2019) Targeting HSP90 with the small molecule inhibitor AUY922 (luminespib) as a treatment strategy against hepatocellular carcinoma. Int J Cancer 144, 2613–2624. 30488605 10.1002/ijc.31963

[28] Li Y , Yin Y , Ma J , Sun Y , Zhou R , Cui B , Zhang Y , Yang J , Yan X , Liu Z and *et al* (2020) Combination of AAV‐mediated NUPR1 knockdown and trifluoperazine induces premature senescence in human lung adenocarcinoma A549 cells in nude mice. Oncol Rep 43, 681–688. 31922247 10.3892/or.2020.7455

